# Natural course of pontocerebellar hypoplasia type 2A

**DOI:** 10.1186/1750-1172-9-70

**Published:** 2014-05-05

**Authors:** Iciar Sánchez-Albisua, Saskia Frölich, Peter G Barth, Maja Steinlin, Ingeborg Krägeloh-Mann

**Affiliations:** 1 Department of Child Neurology, Children's Hospital, University of Tübingen, Hoppe-Seyler-Str. 1, 72072 Tübingen, Germany; 2 Division of Pediatric Neurology, Emma’s Childrens Hospital, Academic Medical Center, University of Amsterdam, Meibergdreef 9, 1105AZ, Amsterdam, The Netherlands; 3 Department of Child Neurology, Children's Hospital “Inselspital”, University of Bern, 3010 Bern, Switzerland

## Abstract

**Introduction:**

Pontocerebellar hypoplasia Type 2 (PCH2) is a rare autosomal recessive condition, defined on MRI by a small cerebellum and ventral pons. Clinical features are severe developmental delay, microcephaly and dyskinesia.Ninety percent carry a p.A307S mutation in the TSEN54-gene. Our aim was to describe the natural course including neurological and developmental features and other aspects of care in a homogeneous group of PCH2 patients all carrying the p.A307S mutation.

**Patients and methods:**

Patients were recruited via the German patients' organizations. Inclusion criteria were imaging findings of PCH2 and a p.A307S mutation. Data were collected using medical reports and patient questionnaires discussed in a standardized telephone interview.

**Results:**

Thirty-three patients were included. When considering survival until age 11 years, 53% of children had died Weight, length and head circumference, mostly in the normal range at birth, became abnormal, especially head circumference (-5.58 SD at age 5 yrs). Neurologic symptoms: Choreathetosis was present in 88% (62% with pyramidal signs), 12% had pure spasticity. Epileptic seizures were manifest in 82%, status epilepticus in 39%. Non-epileptic dystonic attacks occurred in 33%. General symptoms: feeding difficulties were recorded in 100%, sleep disorder in 96%, apneas in 67% and recurrent infections in 52%; gastroesophageal reflux disease was diagnosed in 73%, 67% got percutaneous endoscopic gastrostomy and 36% a Nissen-fundoplication. Neurodevelopmental data: All children made progress, but on a low level: such as fixing and following with the eyes was seen in 76%, attempting to grasp objects (76%), moderate head control (73%), social smile (70%), rolling from prone to supine (58%), and sitting without support (9%). Ten percent lost achieved abilities on follow-up. The presence of prenatal symptoms did not correlate with outcome.

**Conclusion:**

Phenotype of this genetically homogeneous group of PCH2 children was severe with reduced survival, but compatible with some developmental progress. Our data support the hypothesis of an early onset degeneration which thereafter stabilizes.

## Introduction

Pontocerebellar hypoplasias (PCH) are a group of very rare heterogeneous conditions characterized by prenatal development of an abnormally small cerebellum and ventral pons. The main clinical feature is profound psychomotor retardation. In many cases, the disease is fatal early in life. To date, seven types of nonsyndromic PCH have been defined on the basis of clinical and genetic criteria [1]. PCH Type 2 (PCH2) is the most frequently reported - with progressive microcephaly from birth combined with extrapyramidal dyskinesia, and, more rarely, pure spasticity. The inheritance of PCH2 follows an autosomal recessive pattern. Ninety percent of PCH2 cases carry a missense mutation (p.A307S) in the *TSEN54*-gene on chromosome 17, which defines the PCH2A form (OMIM* 608755). Other PCH2 types (PCH2B, PCH2C and PCH2D) are caused by mutations in different genes such as TSEN2, TSEN34 and SEPSECS respectively.

Although the most frequent form of PCH, PCH2A is a rare condition. The estimated incidence is lower than 1:200.000. The disorder is mainly described on a neuropathological, neurostructural (imaging) and genetic level. Descriptions of clinical features tend to summarize symptoms and usually do not include the natural course of the disease. To our knowledge, there is no standardized clinical characterization describing developmental milestones.

However, knowledge of the natural course of the disease is essential for affected families to adjust life perspectives and for physicians to improve prospective management. Also, the natural history of PCH2A could also provide insight into the impact of the gene defect involved and its protein product in relation to brain function and development.

Genes involved in PCH2 are important for protein synthesis during neuronal development [1]. From a neuropathological point of view, PCH2 has been considered a neurodegenerative disorder as in addition to hypoplastic (short cerebellar folia with poor branching) also degenerative changes have been reported [2]. Most degenerative signs, such as sharply demarcated areas where cerebellar cortex lost its full thickness are thought to result from regression at an early stage of development. But neurodegenerative changes thought to happen at a later stage are also reported, such as cystic cerebellar degeneration and vascular changes limited to the cerebellum (such as intimal proliferation and splitting of the elastica interna) found in one patient who died at 22 years [2].

Neurodevelopment in an early onset neurodegenerative disorder is expected to be considerably limited. Two hypotheses are conceivable: on the one hand, cognitive and voluntary motor development are absent. This is how clinical features in PCH2A are usually referred to [1]. On the other hand, single case reports of affected individuals suggest that children may make some neurodevelopmental progress [3]. This would support the hypothesis of an early onset degeneration which thereafter stabilizes and thus would be compatible with some developmental progress.

The aim of this study is to describe the phenotypical variation and natural course in a genetically homogeneous group of children with PCH2A, e.g. all carrying the missense mutation (p.A307S) in the *TSEN54*-gene.

The idea for this study was suggested to us by the PCH2 parents’ organisation in the German speaking countries. They believe that a more thorough and standardized knowledge on their children’s disease course will be helpful for the management of this rare disorder and will also help future patients.

### Patients and methods

Patients were recruited nationwide from April to November 2012 by contacting the families via the PCH2 support group in Germany and Switzerland.

Informed consent was given by the parents in all cases. The study was approved by the ethics committee of the University of Tübingen (no. 105/2012BO2).

*Inclusion criteria* were:

1. Clinical features: primary developmental disorder with dyskinetic/spastic movement disorder

2. Neuroradiology: The imaging findings of PCH, eg. pontocerebellar hypoplasia with pontine and cerebellar hypoplasia affecting cerebellar hemispheres with dragonfly like configuration

3. Genetics: homozygous p.A307S mutation in the *TSEN54* gene

If siblings were clinically affected as described above, they were included on the basis of genetic and imaging findings in the other sibling.

A *standardized questionnaire* was established, covering the essential features of the disease. It included the following items:

1. Age at diagnosis of PCH2A, age at death

2. Family: number of siblings, number of affected siblings, number of deceased siblings, consanguinity of parents, country and region of origin of parents and grandparents

3. Prenatal data: symptoms which have been reported to be associated to PCH (retardation of fetal growth, microcephaly, polyhydramnios or increased fetal movements)

4. Birth: gestational age, multiple birth, birth weight, birth length, head circumference, Apgar score (at age 1, 5 and 10 minutes), admission to a hospital after birth (indication, duration, admission to intensive care unit, duration of mechanical respiratory support)

5. Symptoms (age at onset, age at cessation). Symptoms were defined as follows:

a) Neurological symptoms:

i) Choreoathetoid movement disorder: involuntary, uncontrolled, recurring, and occasionally stereotyped movements. Chorea means rapid involuntary, jerky, often fragmented movements. Athetosis means slower, constantly changing, writhing, or contorting movements.

i) Spastic movement disorder: increased tone, hyperreflexia and Babinski sign resulting in abnormal pattern of movement and posture

i) Non epileptic paroxysmal events/dystonic attacks: sustained dystonic muscle contractions with abnormal, twisted, c-shaped body posture associated to malaise lasting several hours, and EEG without associated epileptic discharges

i) Epileptic paroxysmal events:

ii) seizures: description of epileptic seizures supported by EEG abnormalities

ii) Status epilepticus: convulsive seizures lasting for more than 30 minutes

a) Non-neurological symptoms:

i) Feeding difficulties: incoordination of sucking or swallowing, frequent coughing during meals or a meal lasting longer than 30 minutes

i) Excessive vomiting: vomiting or regurgitation of food once per week or more

i) Excessive sleepiness: the child has to be woken up for meals

i) Recurrent infections: more than 5 episodes of infection-related fever within 6 months

i) Sleep disorder: difficulty falling asleep (restlessness or being awake during the night lasting more than 20 minutes four times a week or more), or difficulty staying asleep (waking up more than once a night more than four times a week).

i) Apneas: cessation of breathing long enough to cause cyanosis and/or pallor

6. Psychomotor development:

As PCH2A is associated with profound psychomotor retardation, we defined very simple abilities following the concept of developmental milestones [4]:

a) Gross motor function:

b) Fine motor function:

i) attempting to grasp objects: directed movements towards an object but without reaching it due to the underlying choreic movement disorder

i) grasping objects: if the objects were clearly reached

i) holding objects: if the objects were reached and kept in hand

b) Language development:

i) ability to produce consistently certain sounds to express approval/disapproval

i) ability to say "yes" or "no" to express approval/disapproval

b) Cognitive skills and communication:

i) ability to recognize familiar people, ability to respond consistently to certain familiar objects (i.e. a toy, a certain ritual, such as the preparations - dressing, etc.- before a walk, a swim or something similar)

i) laughs when talked to

i) fixing and/or following an object or person with the eyes

i) turning the head towards a sound

b) Head control: ability to hold the head for some minutes in prone position or while sitting with support. This was scored as present also if the head control was briefly interrupted by the typical choreic movements of PCH2A.

b) Squirming and rolling

b) Turning from prone to supine, or vice versa

b) Crawling on all four

b) Sitting without support

7. Clinical parameters:

a) Weight, length and head circumference at birth, age 6 weeks, 4 months, 7 months, 1 year, 2 years, 3 years, 4 years, 5 years and current data (evaluated according to growth charts by Voigt and Schneider for length [5], weight and head circumference in the first week of life and the WHO Lenny charts, which include body mass index (BMI), thereafter [6].

b) Surgeries performed and age at procedure: Nissen fundoplication, tracheostomy tube placement, percutaneous endoscopic gastrostomy (PEG) placement.

In addition to the questionnaire, parents were asked for consent that available medical records and MRIs were collected. The questionnaire was sent to the parents and answered with support from one of the authors (SF) in a standardized telephone interview, which lasted between one and two hours. The answers were compared with the medical reports. In case of contradiction, the item was not considered for evaluation. If parental and medical data were consistent, data not explicitly described in the medical records were accepted. All items were answered by the parents. Movement disorders were derived in most cases from medical charts. In the few cases, in which no classification of the movement disorder was available, it was made by three of the authors (ISA, SF and IKM) on the basis of the parents’ description.

### Prenatal symptomatic and prenatal asymptomatic patients

In the literature [3] prenatal onset characterized by the symptoms given above is described to be associated with a more severe disease course (classified as PCH4 and PCH5). Therefore, we were interested in the disease course of children with prenatal signs in comparison to those without.

### Statistical analysis

Quantitative data are presented as mean or median values, standard deviation (SD) and range. Qualitative data between groups were compared by Fisher’s exact test. For quantitative data *t* test was used. Significance was assumed if *p* values were <0.05.

## Results

Data from 34 patients were collected. One child was lacking the genetic results and therefore excluded. A total of 33 patients (17 male; 16 female) with PCH2A, born between 1991 and 2011, were included in the study. All met the clinical criteria and 32 had the typical MRI findings (one clinically equally affected sibling was not imaged). In 31 children, the homozygous p.A307S mutation in the *TSEN54*-gene was found (two more children had clinically equally affected siblings). Four children of our cohort had been included in the publication by Steinlin et al. [7].

Seven families had 2 affected children. There were neither families with more than 2 affected children nor multiple births. There were no consanguineous parents. The country of origin of the grandparents - siblings were counted only once - was known in 101 cases: Germany (n = 78), Switzerland (n = 7), Poland (n = 10) and Russia (n = 6).

### Survival

**Figure 1:**
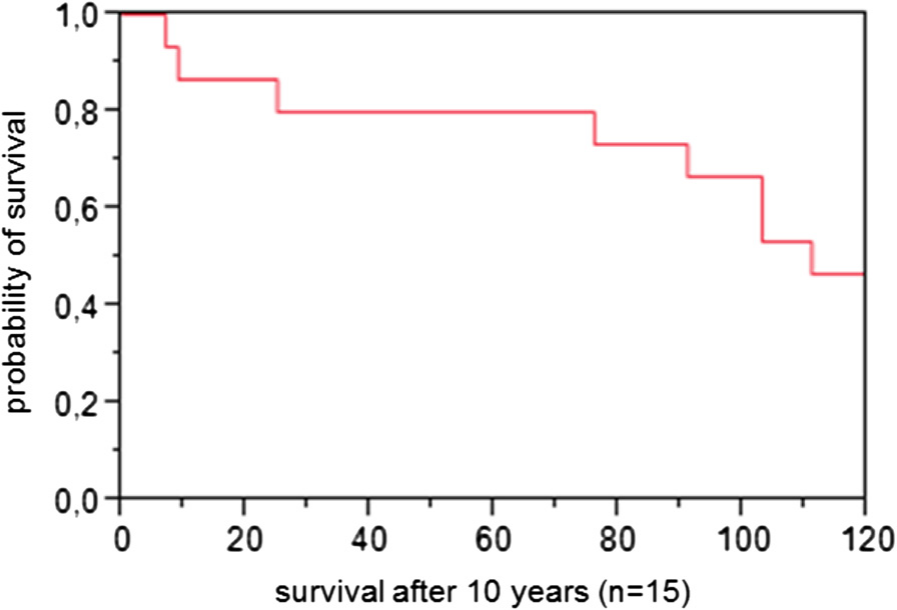
Survival until age 10 years (n=15).

Nine patients (3 girls and 6 boys) had died at the time of the interview. The mean age at the time of death was 6 years and 7 months (SD: 58 months, range: 7 months – 15 years and 6 months). If considering follow-up until the age of 10 years, eight out of 15 children (53%) had died (Figure 1) - three of them before age 6 and five between age 6 and 10. One child had died beyond the age of 10, at 15 years and 6 months. Reported causes and circumstances of death were: sudden unexpected death at night (n = 5), multiorgan failure (n = 1), seizure with apnea (n = 1), pneumonia (n = 1) and hypothermia (n = 1). The latter case concerned a 15 year old child with progressive fall in body temperature which could not be stabilized with medical measures and led to death.

Patients alive at the time of the interview (n = 24) were on average 7 years and 9 months old (SD: 67 months, range: 12 months - 19 years and 6 months): two children were younger than 2 years, eight were between 2 and 5, eight were between 6 and 9, three were between 10 and 15, and three were between 16 and 20. The male:female ratio of patients alive was 1:1,18 (11 boys, 13 girls), respectively.

### Prenatal and neonatal period

*Prenatal signs typically associated to PCH* were reported in 10 children: microcephaly was detected in 4 children by cranial ultrasound at a gestational age between 32 and 35 weeks, in 4 cases there was polyhydramnios, and a total of 6 mothers perceived shaking or trembling fetal movements – which were perceived similar to the abnormal movements after birth.

### Prenatal cranial ultrasonographic findings

In addition to routine ultrasonography – which was performed in 29 cases - a special ultrasound examination of brain and cerebellum was performed in 8 pregnancies. Indication was an affected sibling (n = 4), gestational diabetes (n = 1), advanced maternal age (n = 1), oligohydramnios (n = 1) and unknown (n = 1). It was unrevealing in 7 cases. A posterior fossa fluid collection was seen in one case. In another case, a cranial MRI was carried out in the 21st gestational week and was considered normal except for a cisterna magna size at the upper limit of the normal range. This has already been reported by Steinlin et al. [7].

### Birth and neonatal period

There was one preterm birth in week 36. Umbilical artery pH was in 3 cases below 7.20 (minimum: 7.12). The lowest Apgar score was 6 at 5 minutes (n = 1) and 8 at 10 minutes (n = 1). Birth weight was under the 10th percentile (2420 g) in one case. A total of 20 children (61%) had to be treated for one or several reasons at a Children's Hospital immediately after birth. The average length of stay in hospital was 15 days. Main reasons for admission to hospital in the neonatal period were: respiratory difficulties and/or apnea (n = 9, 27%), feeding difficulties (n = 28, 85%, 13 of them requiring a nasogastric tube), jitteriness (n = 21, 64%), abnormal muscle tone (n = 23, 70%) and excessive sleepiness (n = 19, 58%). Two newborns (6%) required a short-term respiratory assistance. In addition to the symptoms described above, seizures were reported in 2 children and excessive crying in 13.

### Anthropometric measurements

**Figure 2:**
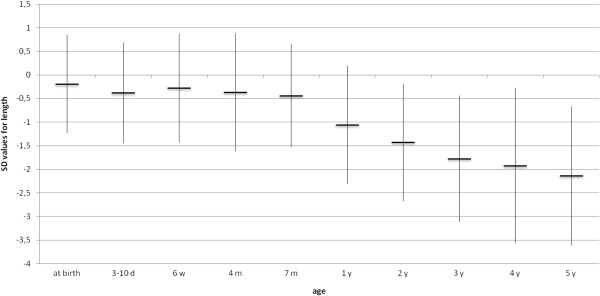
Evolution of mean length (SD) (n=33).

**Figure 3:**
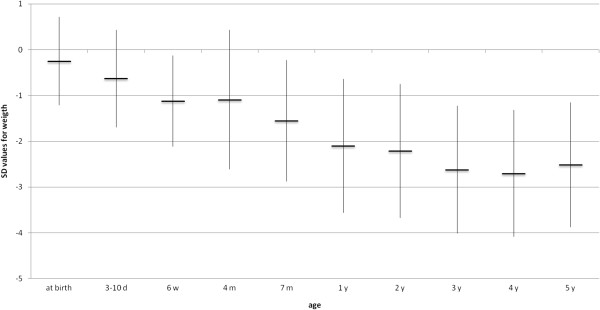
Evolution of mean weight (SD) (n=33).

**Figure 4:**
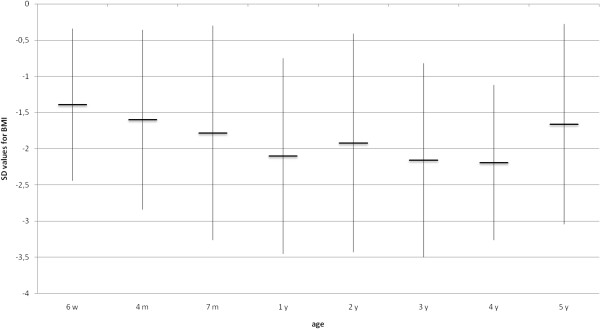
Evolution of mean BMI (SD) (n=33).

**Figure 5:**
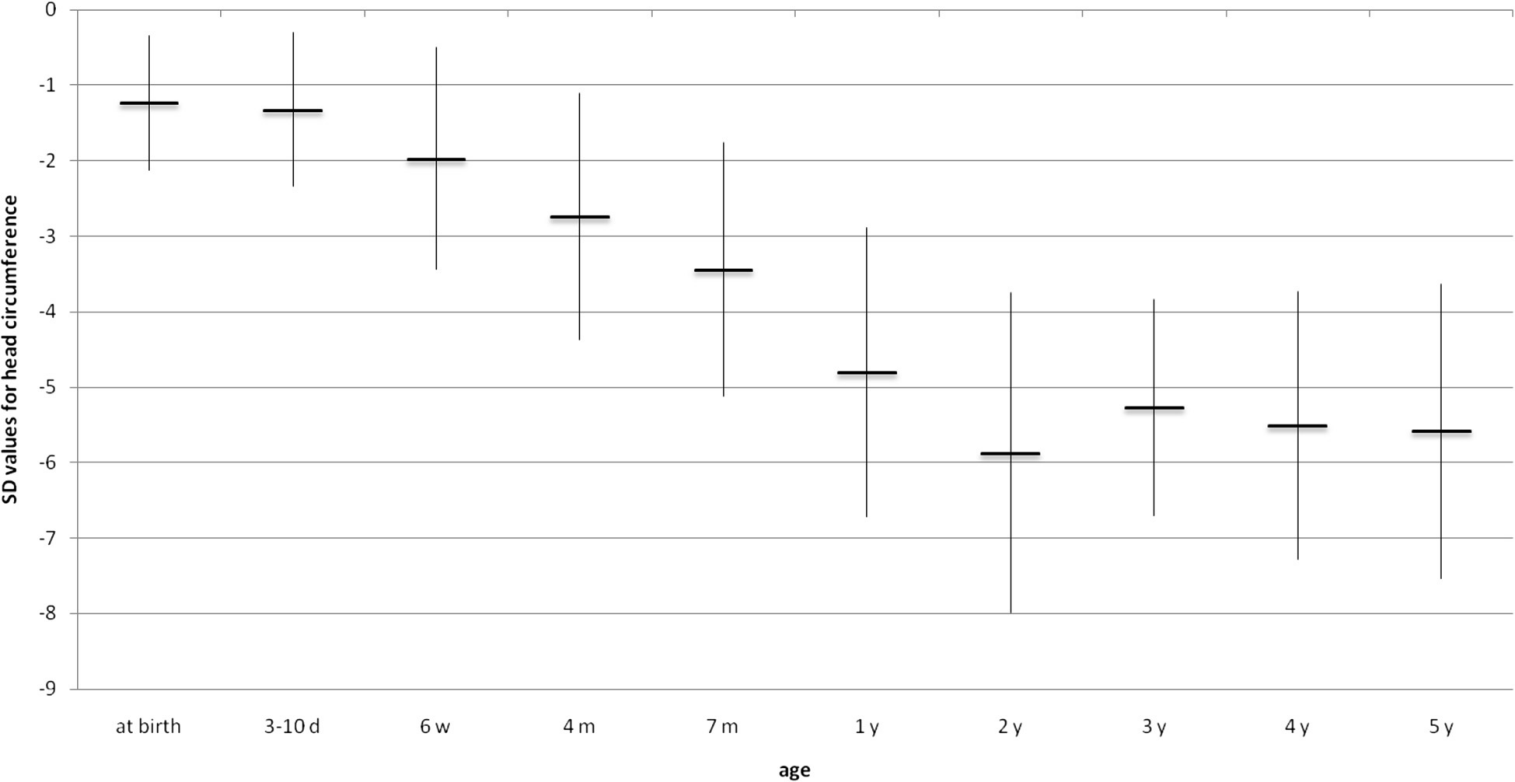
Evolution of mean head circumference (SD) (n=33).

All but 6 (82%) were normocephalic at birth, but all children developed microcephaly during infancy. Four of the six had been diagnosed with microcephaly already during pregnancy. In the other two, microcephaly was evident after birth. Birth weight and birth length were normal in all at birth. As shown in Figures 2, 3, 4, 5, all anthropometric measurements fell below – 2SD on follow up. Especially microcephaly was markedly progressive with increasing age. At birth, it was with a mean value of −1.24 SD in the lower range of normality and at age 5 years the mean SD value of head circumference was −5.58 (range: −2.95 to −8.84), whereas mean length (−2.14 SD) and mean weight (−2.51 SD) were not as extremely abnormal. Interestingly, BMI reached the normal range again at age 5 years.

### Neurological findings

Choreathetoid movements were reported in 29 (88%) patients. They were noticed either immediately after birth or in the first six months of life (average: 3.8 months, SD 4.2). In addition to choreathetosis, 18 of these 29 children had exaggerated tendon reflexes which were in 4 children combined with sustained ankle clonus and a Babinki sign. The other 4 children exhibited a pure spastic movement disorder. Movement disorder persisted in all cases.

### Paroxysmal neurological symptoms

- Eleven children presented with paroxysmal, long-lasting, non-epileptic attacks, which we called "**dystonic attacks"**. Frequency of dystonic attacks was between twice a week and twice a year. During the several hours of the attacks, children adopted an abnormal, twisted, c-shaped body posture. Attacks were associated with malaise and often vomiting.

**Figure 6:**
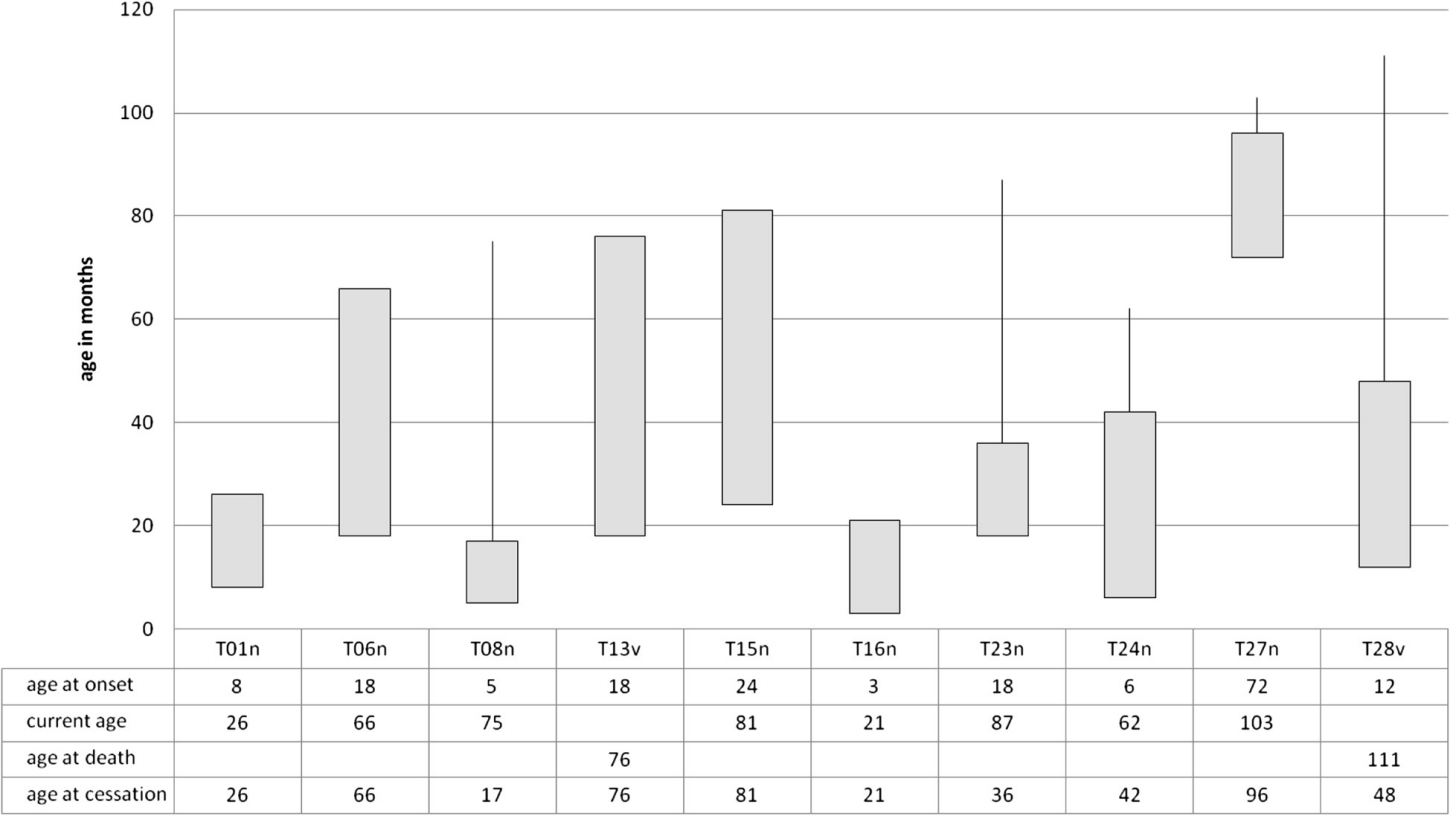
**Onset and duration of dystonic attakcs in 10 children with PCH2.** The bar represents the age (in months) of onset of attacks; if the bar is followed by a line, attacks stopped at that age. One further child is not represented, because age at onset was not known.

Mean age of presentation was 15 months (SD: 19 months, range: 3 months - 6 years) (Figure 6). Dystonic attacks ceased in 5 of 11 affected children between 2 and 8 years of age. The patient not included in the figure, because of unknown age at onset, is currently 19 years old and still suffers from dystonic attacks. In 8 of the 11 children, dystonic attacks only ceased with natural, not drug-induced sleep. Analgesics and L-Dopa were not reported to show benefit. Medication such as diazepam, phenobarbital or chloral hydrate was partially effective in only two children. In two cases, an improvement of dystonic attacks occurred in connection with a dose increase of proton pump inhibitor.

### Seizures

Seizures occurred in 27 of 33 children (82%). The 6 children in whom no seizures were reported were below the age of 28 months. On average, the first seizure occurred at the age of 2 years and 5 months (SD: 28 months, range: 0 months – 9 years).

Patients had frequent (up to several times a day) and different seizure types: The most frequently occurring seizure types were: tonic-clonic (n = 9), atypical absence (n = 9), myoclonic (n = 6), tonic (n = 4), focalized (n = 3), atonic (n = 2), associated with apneas (n = 10).

Many families reported febrile seizures occurring. 25 children were put on antiepileptic drugs, 15 of them combined two or more drugs (Table 1). Seizure freedom was achieved in only 3 cases. If we consider antiepileptic drugs given to more than 5 children, a decrease in seizure frequency in more than half of the patients was reached with phenobarbital and topiramate. The lowest effectiveness was obtained by levetiracetam, which showed a benefit in only 2 out of 8 children.

**Table 1:** Effectiveness of antiepileptic drugs in seizure reduction in 25 children with PCH2A and epilepsy

**Antiepileptic drug**	**Number of children with this medication**	**Reduction in seizure frequency (number of children)**
Carbamazepine	3	2
Clobazam	6	1
Lamotrigine	3	1
Levetiracetam	8	2
Oxcarbazepine	4	1
Phenobarbital	15	11
Sultiam	4	2
Topiramate	7	6
Sodium valproate	6	3
Vigabatrin	3	1

Only antiepileptic drugs given in more than 2 cases are considered.

Characteristic EEG abnormalities were diffuse, abnormal slow activity and multifocal epileptiform discharges. They became evident with increasing age. EEGs in the neonatal period were in nine cases assessed as unremarkable.

Additionally, 39% of patients had **status epilepticus** (13 out of 33). Nine children had one episode, 3 had two and one child had three. The age distribution of the first episode was heterogeneous. On average, the children had their first or only status at age 4 years (46.9 months, SD: 34.6 months, range: 3 months 12 years).

### Neurodevelopmental data

Discrepancies between the answers of the questionnaire and the medical reports were seen in 5 singular answers, which were discarded. It concerned fixing and/or following an object or person with the eyes (n = 3), laughing when talked to (n = 1) and head control (n = 1).

**Figure 7:**
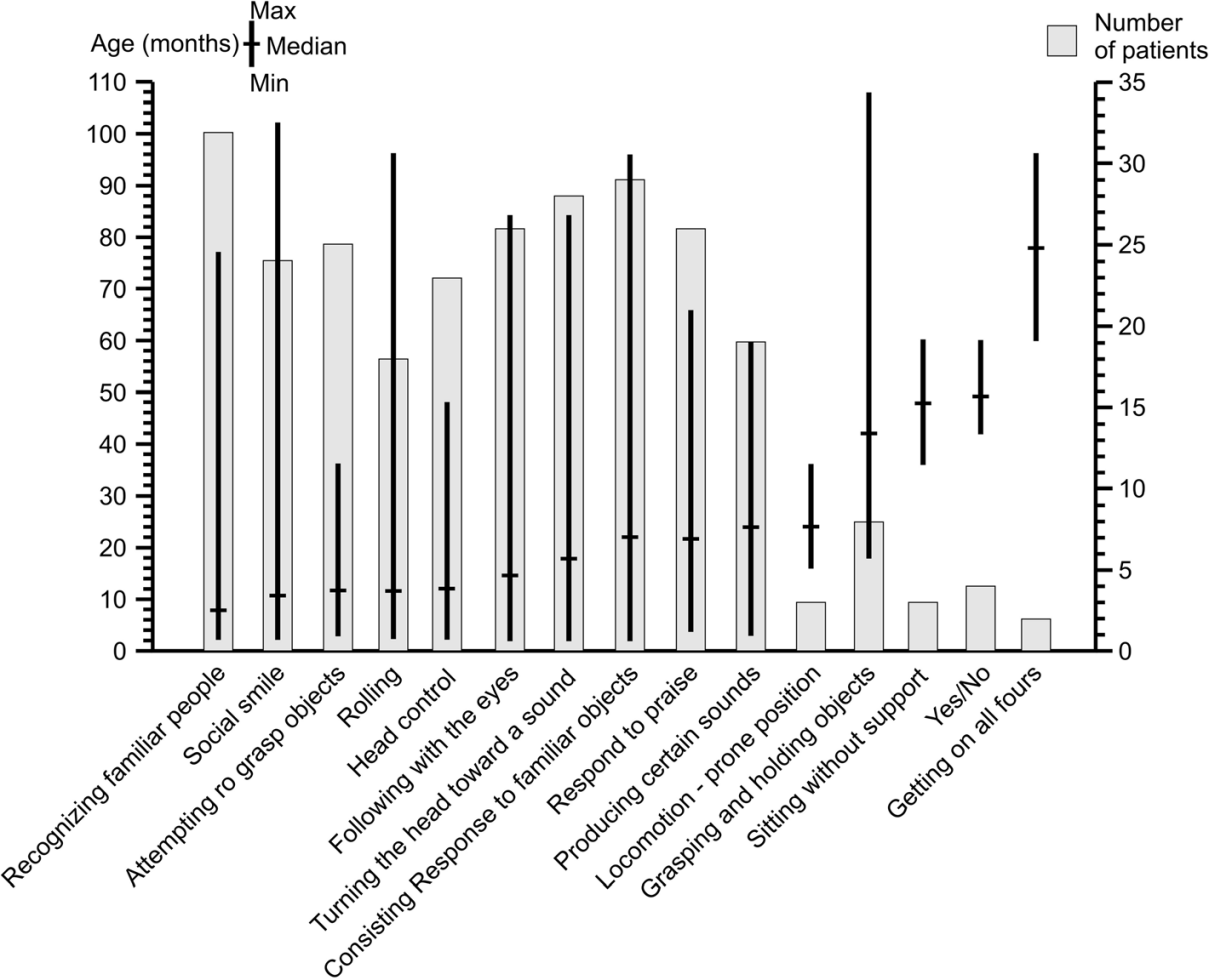
Neurodevelopmental abilities in 33 patients with PCH2A.

As shown in Figure 7, most children reached a partial head control, were able to roll and attempted to grasp objects without reaching them, vocalized to express approval/disapproval, showed a social smile, recognized familiar people and had some visual pursuit. Only few of them were able to get on all fours, sit without support and say "yes" and "no". Data on language abilities were based on the parents' answers in the questionnaire and could not be validated by medical reports. Fixing and following with the eyes was described in medical records only for slow moving objects and for short time intervals.

Once an ability was acquired, it was not lost again in most children. In a few children, some functions were lost subsequently: rolling from prone to supine in 6 out of 19 children aged between 2 and 8 years, sitting without support (one out of 3, achieved at age 3 and lost at age 8,5), attempting to grasp objects (2 out of 25, at age 6.5 and 8), vocalizations (n = 1, at age 9), social smile (1 out of 24, 4%, at age 13.5), following with the eyes (1 out of 24, 4%, at age 6).

Language development was either absent (n = 10) or absolutely rudimentary (n = 19, 56%). In 16 cases, it was reported that joy and well-being could be distinguished from malaise. Three children produced more sophisticated vocalizations according to their parents: a child had different sounds, with which he reacted to different caregivers or with which he expressed hunger and thirst. In addition, another child produced recognizable sounds, accompanied by gestures to express approval or dislike. Four more children were able to produce meaningful words such as “yes” and “no”.

### Non-neurological symptoms

All patients had **feeding problems**. Feeding difficulties started in the first 6 months of life and ceased only in 2 children (6%), who learned to eat small amounts of mushy food with little bits and pieces of foods. The rest of the children were able to swallow at best finely pureed foods (and this with considerable difficulty). A PEG was placed in 22 children at an average age of 3 years and 8 months (SD: 3 years and 4 months, range: 6 months - 14 years).

Excessive vomiting was reported in 31 children (91%). Onset was in the first months of life. It improved in 11 cases at an average age of 4 years and one month (6 months- 8 years and 9 months). Frequency of vomiting was in 6% once a week, in 24% between once a week and once a day and in 64% more than once a day. Gastroesophageal reflux disease was diagnosed in 24 cases. All but one of the 6 children not diagnosed with gastroesophageal reflux had a long history of vomiting. 18 patients were treated with proton pump inhibitors. A Nissen fundoplication was performed in 12 at an average age of 4 years and 7 months (SD: 28 months, range: 9 months- 8 years and 9 months).

A total of 17 (52%) patients had **recurrent infections**, especially upper respiratory tract infections, bronchitis and/or pneumonia (including aspiration pneumonia). One child had, in addition to pneumonias, recurrent urinary tract infections. Infections led to 2 hospitalizations per year.

- **Sleep disorders**: A total of 32 out of 33 (96%) patients exhibited sleep disorders. These started at an average age of 3.7 months (SD: 6.4 months, range: 0–27 months) and improved in 9 cases at an average of 5 years (SD: 35 months, range: 6 months – 12 years). Average time to fall asleep was 70 minutes (SD: 46 minutes, range: 5 – 180 minutes). The mean frequency of nocturnal awakening was 3 times (SD: 1.9 times, range: 0 – 10 times). The only child without sleep disorder was 2 years and 4 months old.

**Apneas** occurred in 22 children (67%), especially at night. Most parents reported a frequency of 2 – 4 apneas per night. Apneas ceased in 5 cases between the age of 8 months and 16 years (median: 42 months). Tactile stimulation was usually sufficient to terminate an isolated apneic event. Thirteen children were monitored using a pulse oximeter, a tracheostomy tube was placed in 5 children at an average age of 7 years (range: 15 months – 14 years).

**Problems in temperature regulation** were reported by many parents, and concerned mainly hyperthermia without evidence of infection (20 children, 60%). Hypothermia was reported only in the child aged 15 (where it led to death), and in one more case immediately after birth.

### Diagnosis

The mean age at diagnosis depended on the year of birth: on average, 6 months for children born after 2008 (SD: 5 months, range: 6 days - 14 months), 12 months for children born between 2003 and 2008 (SD: 28 months, range: 3 days - 120 months), and 5 years for children born before 2003 (SD: 64 months, range: 15 days - 17 years). In 4 cases, the diagnosis was made after death.

### Comparison between prenatal symptomatic and prenatal asymptomatic patients

Survival rate in the prenatal symptomatic and prenatal asymptomatic group differed neither at age 1 (8 out of 10 *vs.* 22 out of 22, *p* = 0.09), nor at age 5 (7 out of 9 *vs*. 16 out of 17, *p* = 0.3), nor at age 10 (3 out of 7 *vs*. 4 out of 8, *p* = 1).

The groups did not differ with respect to symptoms in the neonatal period. There was no difference neither in the frequency of admission to hospital between prenatal symptomatic and asymptomatic patients (70% *vs.* 56%, *p* = 0.7), nor in average length of stay in hospital (19 vs. 13 days, *p* = 0.1) nor concerning need for respiratory support (n = 1 in both groups).

In the postneonatal period, frequency of dystonic attacks, seizures, constipation, recurrent infections, sleep disorders and apneas was similar in both groups. Neurodevelopmental milestones were achieved by a similar proportion of children of both groups at a similar age. The only exception was the ability to recognize familiar objects, which was more frequent in the prenatal asymptomatic patients (22 out of 23 [95.7%] *vs.* 7 out of 10 [70%], *p* = 0.04).

## Discussion

We investigated the natural course in a cohort of 33 children with PCH2A using a standardized questionnaire and medical records. PCH2 is a very rare disease, less than 100 families have been reported so far [1,8]. In the Dutch and German population the carrier frequency of the responsible mutation is 0.004 [1]. PCH2 is a severe condition with high mortality in childhood and profound disability [3]. We wanted to know whether the condition is characterized by a lack of any neurodevelopmental progress and increasing burden of multi-morbidity or whether some developmental milestones can be achieved despite severe multimorbidity, informations which are crucial for affected families. Inclusion criteria were clinical and MRI findings along with a pathogenic mutation. We restricted our inclusion criteria to homozygosity of the p.A307S mutation in the *TSEN54*-gene (also called “common mutation”, which defines the PCH2A form) for several reasons: first, it is the most common mutation and responsible for 90% of PCH2 cases; second, in centering on a homogeneous genotype, a detailed phenotyping will shed light on the effects of the gene defect involved.

We could characterize PCH2A concerning the disease course and neurodevelopment with respect to the following domains

Survival and prenatal onset

Anthropometric measures

neurological symptoms including paroxysmal events

developmental progress

additional morbidity – feeding and sleeping problems.

*Life expectancy* was considerably reduced in our cohort. Most patients did not reach puberty, consistent with what has been reported in another series of 16 patients [9]. As *prenatal signs* such as polyhydramnios, microcephaly and abnormal fetal movements (shaking, trembling movements) are considered features of the more severe forms of PCH, PCH4 and PCH5 [1], we compared the course of children showing these signs to those who did not. Prenatal signs were found in ten out of the 33 children, and they did differ from prenatally asymptomatic children neither concerning survival nor perinatal complications, postnatal symptoms nor anthropometric measures. Neurodevelopment was also similar in both groups. Thus, at least in children carrying the mutation (p.A307S) in the *TSEN54*-gene, prenatal symptoms do not seem to be negative predictors of postnatal severity.

*Anthropometric measures* were mostly within the normal range at birth. A markedly progressive microcephaly is known to become more evident with increasing age [3,7]. We could quantify the microcephaly to fall below 5 SDs whereas both failure to thrive and short stature were not as severe, falling below 2 SDs.

### Neurological symptoms including paroxysmal events

Nearly 90% of patients showed a predominant choreathetotic movement disorder present in the first months of life, only a few had pure spasticity. This is consistent with the first description by Barth [9].

Seizures were very frequent and heterogeneous, even in the same patient. EEG abnormalities in all were diffusely abnormal slow rhythms and multifocal epileptiform discharges. The risk of status epilepticus was high. The epilepsy was drug resistant in most cases. Seizures types and EEG abnormalities have not been systematically described in previous studies. Only Grosso et al. described generalized tonic-clonic seizures in a case report [10]. In the study of Steinlin et al. [7], only half of PCH2 children (n = 24) were reported with epilepsy, which could be due to a younger age at assessment (not reported).

To our knowledge, the symptom "dystonic attacks" has not been systematically described. Grosso et al. [10] reported on one child with what they called ‘dystonic crises’ which could correspond to the feature we describe, as they report repetitive dystonic posturing of one leg in extension associated with irritability and crying. In our study, one third showed such attacks, the appearance of which was uniform: dystonic posturing with a c-shaped posture for several hours accompanied by malaise and often vomiting, without associated epileptiform discharges on EEG. Attacks ceased with age in half of the affected children. They seem difficult to treat as medication was not reported effective in our cohort. In most cases, they only ceased with natural, not drug-induced sleep. The pathomechanism is unknown. Interestingly, in 2 children they improved after increasing the dose of a proton pump inhibitor, which might suggest a connection to gastroesophageal reflux.

### Developmental progress

PCH2A is associated with profound disability [3,7]. In our cohort, no patient ever achieved the milestones of crawling, standing, walking or talking. Visual fixation was persistently poor. Nevertheless, around two third of children made some progress, although on a very low level: they gained partial head control, were able to roll and attempted to grasp objects without reaching them, vocalized to express approval/disapproval, showed a social smile, recognized familiar people and had some visual pursuit. A few could even sit without support and get on all fours, in contrast to previous reports [7]. Some functions such as grasping objects seemed impaired not only because of the cognitive impairment, but due to the dyskinetic movement disorder.

Interestingly, only a few patients lost the functions they had achieved, although PCH2A has been considered a neurodegenerative disorder [2]. PCH2A is due to mutations in three tRNA splicing endonuclease (TSNE) subunit genes. Intron containing tRNAs require the TSEN complex for splicing of tRNA, which is an essential step in protein synthesis [11]. These processes are important in developing neurons. It has been suggested that there is a time frame during embryogenesis in which there is an extra high demand for protein synthesis in neuronal tissue in the early post-migratory stage which could explain an early degeneration with some stabilization thereafter [1]. Our results support this hypothesis of an early onset degeneration leading to severe pontocerebellar maldevelopment and insufficient brain growth which may, however, stabilize thereafter and is compatible with some developmental progress. The severe microcephaly during development - which is much more marked than the deficit of length growth – may have two explanations: 1) the cerebellum establishes millions of projections to the telencephalon, especially to the frontal lobe [12] during fetal and early infantile life. PCH most certainly disturbs this development. 2) On the other hand, an ongoing neurodegeneration may also play a role.

### Additional morbidity –feeding and sleeping problems

All patients exhibited early abnormalities. Feeding difficulties (sucking or swallowing difficulties, excessive vomiting) had already been described [3]. They are most likely due to both bulbar dysfunction and gastroesophageal reflux. In our cohort, gastroesophageal reflux disease was diagnosed or could be clinically suspected in almost all children. We think, it is an important observation, which could encourage treating doctors to recognize and treat this condition very early.

Sleeping problems have not been previously reported, but were present in virtually all patients of our cohort. Both average time to fall asleep and frequency of nocturnal awakenings were affected.

The frequency of apneas per night was based on the parents’ reports and might be underdiagnosed, as only 13 out of 22 children in which apneas were described were monitored using a pulse oximeter. Parents may not be able to recognize every single apnea during a night. Actually, apnea might be a common cause of death in our cohort: the parents reported in 5 cases sudden unexpected death and in one case seizures with apnea.

In **conclusion**, our group of patients with PCH2A - genetically homogeneous, all carrying the homozygous missense mutation (p.A307S) in the *TSEN54*-gene – showed a severe, but nevertheless variable phenotype. Survival was reduced and most patients did not reach puberty. Anthropometric measures, within the normal range at birth, showed markedly progressive microcephaly falling below 5 standard deviations from the mean already in the second year of life, whereas both failure to thrive and short stature only fell below 2 SDs. Neurological signs were characterized mainly by choreoathetosis. Epileptic seizures were prominent and difficult to treat. A feature described for the first time were dystonic attacks occurring in one third of patients and lasting for several hours which seemed to be painful; onset was in the first two years of life and attacks often persisted for years. In addition, feeding problems requiring PEG and Nissen-fundoplication, sleep disorders, apneas, and recurrent infections contributed to severe multi-morbidity. Despite this, around two third of the patients made some developmental progress in both cognitive and motor domains. Interestingly, the presence of prenatal symptoms did not correlate with a poorer prognosis.

## Competing interests

The authors declare that they have no competing interests.

## Authors’ contributions

ISA, SF and IKM conceived of the study, established the questionnaire, and participated in the design and drafted the manuscript. SF recruited the patients by contacting the families via the PCH2 support group in Germany and Switzerland. She also performed the telephone interviews. MS and PB critically revised the manuscript. MS was also involved in patient recruitment. All authors read and approved the final manuscript.
